# lncRNA JPX/miR-33a-5p/Twist1 axis regulates tumorigenesis and metastasis of lung cancer by activating Wnt/β-catenin signaling

**DOI:** 10.1186/s12943-020-1133-9

**Published:** 2020-01-15

**Authors:** Jinchang Pan, Shuai Fang, Haihua Tian, Chengwei Zhou, Xiaodong Zhao, Hui Tian, Jinxian He, Weiyu Shen, Xiaodan Meng, Xiaofeng Jin, Zhaohui Gong

**Affiliations:** 1 Department of Biochemistry and Molecular Biology, Ningbo University School of Medicine, Ningbo, 315211 China; 2 Zhejiang Province Key Laboratory of Pathophysiology, Ningbo University School of Medicine, Ningbo, 315211 China; 3 Department of Laboratory Medicine, The Affiliated Ningbo Kangning Hospital of Ningbo University School of Medicine, Ningbo, 315201 China; 4 Department of Thoracic Surgery, The Affiliated Hospital of Ningbo University School of Medicine, Ningbo, 315020 China; 5 Department of Thoracic Surgery, The Affiliated Ningbo Medical Center Lihuili Eastern Hospital of Ningbo University School of Medicine, Ningbo, 315048 China

**Keywords:** Epithelial-mesenchymal transition, Twist1, Long noncoding RNA, Wnt/β-catenin signaling, Lung cancer

## Abstract

**Background:**

MicroRNAs (miRNAs) and Twist1-induced epithelial-mesenchymal transition (EMT) in cancer cell dissemination are well established, but the involvement of long noncoding RNAs (lncRNAs) in Twist1-mediated signaling remains largely unknown.

**Methods:**

RT-qPCR and western blotting were conducted to detect the expression levels of lncRNA JPX and Twist1 in lung cancer cell lines and tissues. The impact of JPX on Twist1 expression, cell growth, invasion, apoptosis, and in vivo tumor growth were investigated in lung cancer cells by western blotting, rescue experiments, colony formation assay, flow cytometry, and xenograft animal experiment.

**Results:**

We observed that lncRNA JPX was upregulated in lung cancer metastatic tissues and was closely correlated with tumor size and an advanced stage. Functionally, JPX promoted lung cancer cell proliferation in vitro and facilitated lung tumor growth in vivo. Additionally, JPX upregulated Twist1 by competitively sponging miR-33a-5p and subsequently induced EMT and lung cancer cell invasion. Interestingly, JPX and Twist1 were coordinately upregulated in lung cancer tissues and cells. Mechanically, the JPX/miR-33a-5p/Twist1 axis participated in EMT progression by activating Wnt/β-catenin signaling.

**Conclusions:**

These findings suggest that lncRNA JPX, a mediator of Twist1 signaling, could predispose lung cancer cells to metastasis and may serve as a potential target for targeted therapy.

## Background

Lung cancer is one of the most malignant of all cancer, and the 5-year survival rates vary from 4 to 17% depending on stage and regional differences [1, 2]. Although many advances have been made in the diagnosis and treatment of lung cancer in recent years, metastasis still remains the main challenge posed by advanced lung cancer leading to high mortality [3]. Thus, the elucidation of a new oncogenic pathway is required to precisely target lung cancer and to serve as a prognostic factor. Long noncoding RNAs (lncRNAs) are a class of RNA molecules longer than 200 nucleotides in length with considerable potential to drive cancer development [4–7].

Aberrantly expressed lncRNAs have been found to be associated with the occurrence and development of various types of cancers [8–10]. In addition, lncRNAs affect gene expression through various mechanisms in which lncRNAs regulate their target genes by acting as microRNA (miRNA) sponges, thereby affecting the growth, proliferation, migration, and invasion of cancer cells [11, 12]. In the lncRNA-miRNA-mRNA regulatory network, lncRNAs act as competitive endogenous RNAs (ceRNAs) of specific mRNAs [13]. Specifically, lncRNA SMAD5-AS1 could upregulate adenomatous polyposis coli expression by sponging miR-135b-5p and inactivate the canonical Wnt/β-catenin pathway to inhibit diffuse large B cell lymphoma proliferation [14]. Furthermore, lncRNA PVT1 was found to regulate hexokinase 2 (HK2) expression by competitively binding to endogenous miR-143 in gallbladder cancer (GBC) cells, suggesting an important role of the PVT1/miR-143/HK2 axis in cell proliferation and metastasis by modulating aerobic glucose metabolism in GBC cells [15]. However, the function and mechanism of most aberrantly expressed lncRNAs as ceRNAs in lung cancer remain unclear.

Our previous work showed that miR-33a-5p negatively regulated Twist1, thus inhibiting the invasion and metastasis of non-small cell lung cancer (NSCLC) [16], and served as a potential biomarker for early lung cancer diagnosis [17]. Twist1 is an important transcription factor that mediates epithelial-mesenchymal transition (EMT) progression and tumor metastasis [18, 19]. In addition, Wnt/β-catenin signaling is a critical driver in EMT and cancer metastasis [20]. It has been found that miRNAs can participate in the EMT process by regulating the Wnt/β-catenin pathway in a variety of cancers [21, 22]. Therefore, we considered whether an lncRNA could form a ceRNA network with miR-33a-5p and Twist1 to participate in EMT and malignant processes in lung cancer. In the present study, we identified that JPX, an upregulated lncRNA in lung cancer, acted as a ceRNA for Twist1 through binding with miR-33a-5p. As an oncogene, JPX affected the tumor size, TNM staging, and metastasis of lung cancer. Functionally, JPX promoted cell proliferation, migration, and invasion and facilitated tumor growth in xenograft mouse model. Further assays revealed that JPX participated in the activation of Wnt/β-catenin signaling by regulating miR-33a-5p/Twist1, which in turn promoted the EMT process, ultimately influencing the of lung cancer process. The results indicate that the JPX/miR-33a-5p/Twist1 axis regulates lung carcinoma by activating Wnt/β-catenin signaling, suggesting a therapeutic potential for lung cancer treatment.

## Methods

### Clinical subjects and specimens

Total of 116 lung cancer tissues and corresponding adjacent tissues were collected from the Affiliated Hospital of Medical School of Ningbo University (Ningbo, China) and Ningbo Medical Center Lihuili Eastern Hospital (Ningbo, China). All of the patients were diagnosed with primary lung cancer and did not receive preoperative radiotherapy, chemotherapy, targeted therapy and immunotherapy. At the same time, general clinical information and detailed pathology records were collected. All patients received written informed consent and the study protocol was approved by the Clinical Research Ethics Committee of the Medical School of Ningbo University (Approval No.: NBUSM20171006). All experimental protocols were implemented in accordance with relevant regulations.

### RNA extraction and quantitative real-time PCR (RT-qPCR)

Total RNA was isolated from lung cancer tissue and cells using Trizol reagent (Invitrogen, USA) following the manufacturer’s protocol. DeNovix DS-11 Spectrophotometer (DeNovix, USA) detects the purity and concentration of RNA. We synthesized cDNA by reverse transcription reaction using a ReverTra Ace qPCR RT Master Mix with gDNA Remove kit (Toyobo, Japan) or a commercial miRNA reverse transcription PCR kit (GenePharma, China). RT-qPCR was conducted using a SYBR Premix Ex Taq II (Takara, Japan) on a Mx3005P real-time PCR System (Stratagene, USA) according to manufacturer’s instructions. Results were normalized using glyceraldehyde-3-phosphate dehydrogenase (GAPDH) or U6 as an internal control. To account for the assessment of technical variability, the assays were performed in triplicate for each case. Primer sequences are shown in Additional file 1: Table S1.

### Cell culture and transfection

All cell lines were obtained from the Chinese Academy of Sciences Cell Bank (CASCB, China), including 1 human normal bronchial epithelial cell (BEAS-2B) and 4 human lung adenocarcinoma cells (SPC-A-1, LTEP-a-2, A549, NCI-H1299). All human lung cancer cell lines were cultured in RPMI-1640 (Hyclone, USA), with 10% fetal bovine serum (PAN, Germany). BEAS-2B was maintained in Dulbecco’s modified Eagle’s medium (DMEM) that was supplemented with 10% FBS. All cell lines were placed in a cell culture incubator (Thermo Fisher, USA) containing 5% CO_2_ at 37 °C. JPX small interfering RNA (siRNA, GenePharma, China) and *Twist1* siRNA with the corresponding control RNA (siRNA NC), or recombinant plasmid overexpressing JPX with the empty pcDNA3.1 vector (Tiandz, China), or miR-33a-5p mimics (GenePharma, China) with corresponding control RNA (mimics NC) were transfected into cells in logarithmic growth phase. The transfection was performed using the Lipofectamine 2000 transfection reagent (Invitrogen, USA) according to the manufacturer’s protocol. The transfected sequences of the miR-33a-5p mimics and siRNA oligonucleotides are shown in Additional file 1, Table S2.

### Recombinant plasmid construction

The sequences of JPX was amplified by PCR from the genomic DNA of SPC-A1 cell line, and sub-cloned into the pcDNA3.1 vector or pGL3-control vector (Promega, USA) as described in our previous work [16]. The primer sequences are shown in Additional file 1, Table S1.

### Cell counting Kit-8 (CCK-8) assay

The transfected cells were seeded in 96-well plates at a concentration of 5 × 10^3^ per well at different time points (24, 48, 72, and 96 h), and 10 ml CCK-8 reagent (Dojindo, Japan) was added to each well after cell attachment, and cells were incubated at 37 °C for 2 h. We determined the cell growth rate by measuring their optical density (OD) value at 450 nm using a microplate reader (Labsystems, Finland).

### Colony formation assay

The transfected cell suspension was collected, and 500 cells were seeded ito a 6-well plate and cultured in a cell culture incubator. After 2 weeks, the cell colonies were washed 3 times with 1 × PBS. Colonies were fixed with 4% paraformaldehyde for 30 min and stained with 0.1% crystal violet (Solarbio, China) for 30 min.

### Wound healing assay

The confluent cell monolayer was manually damaged by scraping the cells with a 200 μl pipette tip. Photographs were taken using an optical microscope (Olympus, Japan) at 0, 24, and 48 h, respectively. The distances were measured by Image-Pro Plus 6.0 software.

### Transwell invasion assay

The transfected cells were collected and resuspended in serum-free medium. Then, 1 × 10^5^ cells were seeded into a pre-packed Matrigel (BD Bioscience, USA) chamber (Corning, USA), and the chamber was inserted into a well containing 20% serum from 24-well plate. After 24 h incubation, the cells remaining on the upper membrane surface were removed using a cotton swab, and the cells adhering to the lower membrane surface were fixed with 4% paraformaldehyde and stained with 0.1% crystal violet. Cells were then counted under an optical microscope.

### Nuclear and cytoplasmic RNA fractionation analysis

Nuclear and cytosolic fractions were separated using a PARIS kit (Thermo Fisher Scientific, USA) according to the manufacturer’s instruction. The expression levels of GAPDH, U6 and JPX in the nuclear and cytoplasm of lung cancer cells were detected by RT-qPCR assays.

### Cell lysates and western blotting

We extracted the protein (including total, nuclear and cytoplasmic protein) of the cells using RIPA lysis buffer (50 mM Tris-HCl pH 8.0, 150 mM NaCl, 1%Triton X-100, and 1 protease inhibitor cocktail tablet/10 ml) and detected the protein concentration with a BCA kit (Beyotime, China). The western blotting was conducted as previously described [23]. The primary antibodies were anti-E-cadherin (Bioss, USA), anti-N-cadherin (Santa Cruz, USA), anti-Vimentin (CST, USA), anti-GSK-3-β (Bioss, USA), anti-β-Catenin (CST, USA), anti-Twist1 (Sigma, USA), anti-GAPDH (Santa Cruz, USA), and anti-Lamin B (Bioss, USA).

### Bioinformatic analysis

The putative miRNA binding sites on JPX sequences were predicted using StarBase V3.0 (http://starbase.sysu.edu.cn/).

### Luciferase reporter assay

JPX wild-type and mutant-type luciferase reporter vector targeting the miR-33a-5p binding site were constructed. The vectors and miR-33a-5p mimics were co-transfected into cells by Lipofectamine 2000 reagent, and luciferase activities were measured 24 h later using the dual luciferase reporter system (Promega, USA). Renilla luciferase activity was used as a standardized control.

### In vivo tumorigenesis assay

Four-week-old BALB/c male nude mice were purchased from Shanghai SLAC Laboratory Animals Co., Ltd. (Shanghai, China). We screened lung cancer cells stably expressing JPX and empty plasmids using G418 and stably expressed miR-33a-5p by transfecting AgomiR-33a-5p (GenePharma, China). AgomiRNA is a specially labeled and chemically modified double-stranded small RNA that mimics the endogenous miRNA to regulate the biological function of the target gene. The subcutaneous xenograft mouse model was used to assess the tumor formation ability. First, 5 × 10^6^ lung cancer cells (SPC-A-1 and NCI-H1299) stably expressing miR-NC/miR-33a-5p, and empty plasmid/JPX were suspended in 200 μL phosphate-buffered saline (PBS) and then were injected subcutaneously into the right flanks of BALB/c nude mice (single-factor experiment, *n* = 7 per group; rescue experiment *n* = 3 per group). The tumor dimensions were measured every two days via digital caliper measurements; after 4 weeks, the mice were sacrificed by cervical dislocation and the tumors were excised for weighing. The tumor volume was calculated with the formula V = (length × width^2^)/2. To determine lung cancer cell metastasis, SPC-A-1 and NCI-H1299 cells stably expressing miR-NC/miR-33a-5p, and empty plasmid/JPX were used to construct a tail vein metastasis animal model. Each BALB/c nude mouse was injected with 200 μL PBS containing 5 × 10^6^ cells (*n* = 3 each group). All nude mice were sacrificed humanely after 30 days, and intact lung tissues were obtained f and imaged. The tissue sections were used for subsequent experiments, such as H&E staining. To prevent AgomiR-33a-5p degradation in vivo, we injected a dose of 1 nmol AgomiR-33a-5p (in 20 μL PBS) into the subcutaneous tumors and tail veins of nude mice weekly. The experiments were performed in accordance with the approved guidelines of the Laboratory Animal Ethical Committee at Ningbo University (Approval No.: NBULA20180902/ NBULA20191109).

### Immunohistochemistry assay (IHC)

The paraffin-embedded tumor tissue sections were deparaffinized and rehydrated for IHC, and the antigen was retrieved with high pressure in 0.01 M sodium citrate buffer solution. After incubating with the primary and secondary antibodies, the sections were incubated with diaminobenzidine and counterstained with hematoxylin (Solarbio, China). Images were taken by a microscope with 200× magnification (Olympus, Japan). Primary antibody for IHC: anti-Twist1 (Bioss, USA), anti-β-catenin (Bioss, USA).

### Statistical analysis

The statistical analyses were carried out with using GraphPad Prism 8 software. Data are presented as the mean ± SD, and all experiments were performed in triplicate. The relationship between JPX expression and the clinical characteristics of patients with lung cancer were evaluated using the chi-squared test. Analysis of differences between the two groups were performed using Student’s t test, one-way ANOVA, and Pearson’s correlation analysis. For all analyses, a *P*-value less than 0.05 from a two-tailed test was considered statistically significant.

### Data availability

The data that support the findings of this study are available from the corresponding author upon reasonable request.

## Results

### JPX was upregulated in lung cancer tissues and cells

**Fig. 1:**
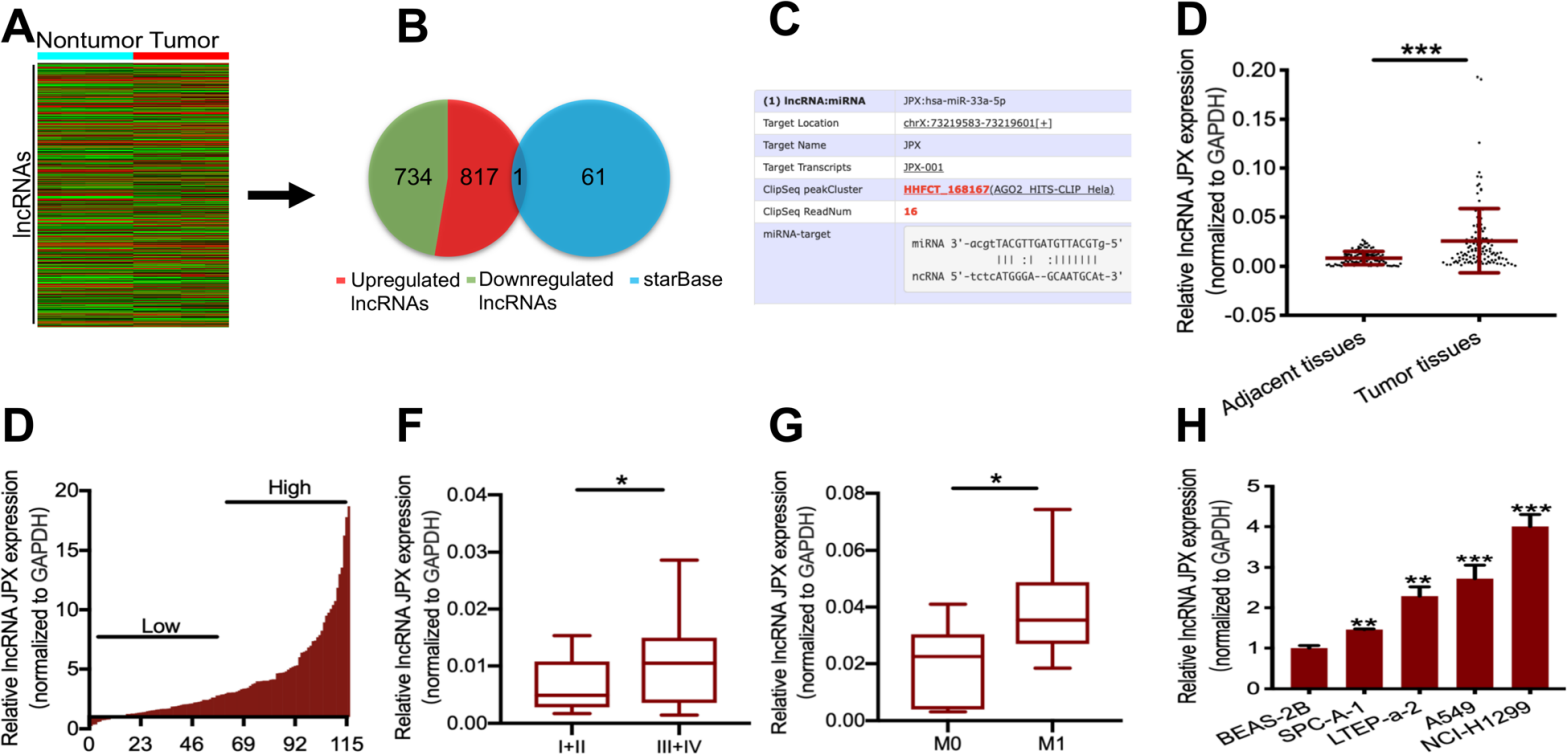
JPX was ectopically overexpressed in lung cancer tissues and cells. (**a**) The heatmap of differentially expressed lncRNAs from four pairs of lung cancer tissues and matched adjacent normal tissues. The red color on the side of the row represents the upregulated lncRNAs in lung cancer, while the green color represents downregulated lncRNAs. (**b**) Venn diagram showing overlapping lncRNAs from the results of lncRNA microarray analysis and starBase database prediction. (**c**)Targeted sites between miR-33a-5p and JPX in starBase database. (**d**)Relative JPX expression detected by RT-qPCR in 116 paired lung cancer and noncancerous tissues. Results are presented as 2^-ΔΔCt^ in tumor tissues relative to normal tissues. (**e**)Relative JPX expression of in lung cancer tissues was divided into 58 high-expression groups and 58 low-expression groups according to the median. (**f, g**)Relative JPX expression in lung cancer tissues with different TNM stages and with or without metastasis. (**h**)RT-qPCR analysis of the relative JPX expression in four lung cancer cell lines (SPC-A-1, LTEP-a-2, A549, NCI-H1299) and a normal lung epithelial cell line (BEAS-2B). Data are presented as the mean ± SD from three independent experiments. *P < 0.05, **P < 0.01, ***P < 0.001

We performed lncRNA microarray analysis to identify differentially expressed lncRNAs using four pairs of human lung cancer tissues and matched nontumor tissues. In total, 1551 differentially expressed lncRNAs were identified, of which 817 lncRNAs were highly expressed and of which 734 were lowly expressed (Fig. 1a and b). Our previous work showed that miR-33a-5p could inhibit lung cancer invasion and metastasis [16]. Here, we expected to obtain differentially lncRNAs that bind with miR-33a-5p. The miRNA-lncRNA interaction database (http://starbase.sysu.edu.cn/starbase2/index.php) was used to predict the potential lncRNAs that could interact with miR-33a-5p. The results showed that 61 lncRNAs shared binding sites with miR-33a-5p. The overlapping analysis of 1551 differentially expressed lncRNAs and the predicted 61 lncRNAs indicated that only lncRNA JPX interacted with miR-33a-5p via seven consecutive pairings of complementary base pairs (Fig. 1b and c). To confirm JPX expression in lung cancer tissues, we performed RT-qPCR to detect JPX in 116 pairs of lung cancer tissues and adjacent noncancerous tissues and found significantly higher JPX expression in lung cancer tissues than in adjacent normal tissues (Fig. 1d). To further investigate the association between the JPX expression and the clinicopathological characteristics, 116 human lung cancer tissue samples were divided into two subgroups according to the median ratio of relative JPX expression: the high JPX group (*n* = 58) and the low JPX group (n = 58) (Fig. 1e). Correlation regression analysis showed that the high JPX expression in 116 lung cancer patients was closely related to large tumor size (*P* = 0.0009) and the advanced TNM stage (*P* = 0.0003). However, gender (*P* = 0.7077), age (*P* = 0.4346), smoking history (*P* = 0.3443), histology (*P* = 0.9042), tumor location (*P* = 0.193) and differentiation (*P* = 0.8095) were not correlated with JPX expression (Table 1). In addition, JPX was expressed more frequently in advanced and metastatic lung cancer patients than that in early and non-metastatic lung cancer patients (Fig. 1f and g). Next, we measured JPX expression in human normal lung bronchial epithelial cells (BEAS-2B) and four lung cancer cell lines (SPC-A-1, LTEP-a-2, A549, NCI-H1299) by RT-qPCR. As shown in Fig. 1h, JPX expression in these four cell lines was significantly higher than that in the normal lung bronchial epithelial cells. Among the four cell lines, relative JPX expression was the lowest in SPC-A-1 cells and highest in NCI-H1299 cells. Therefore, SPC-A-1 and NCI-H1299 cell lines were selected as subjects for the subsequent cell phenotype assays. Together, the results suggested that JPX was aberrantly upregulated in lung cancer tissues and cells and was closely correlated with tumor size and TNM stage, suggesting an oncogenic role of JPX in lung cancer.

**Table 1:** Correlations between JPX and clinical characteristics of 116 lung cancer patients

	n	lncRNA JPX level^†^		χ^2^ test	*P* value
Characteristics		High	Low		
Total cases	116	58	58		
Gender					
Male	66	34	32	0.1406	0.7077
Female	50	24	26		
Age (years)					
≤ 60	40	18	22	0.6105	0.4346
>60	76	40	36		
Smoking history					
Yes	47	26	21	0.8942	0.3443
No	69	32	37		
Histology					
Adenocarcinoma	84	41	43	0.2015	0.9042
Squamous carcinoma	26	14	12		
Other type	6	3	3		
Tumor location					
Left	55	24	31	1.694	0.193
Right	61	34	27		
Differentiation					
High and moderate	21	11	10	0.05815	0.8095
Poor	95	47	48		
Tumor size(cm)					
≤ 3	84	34	50	11.05	0.0009^**^
>3	32	24	8		
TNM stage					
I + II	87	35	52	13.29	0.0003^***^
III + IV	29	23	6		

†Median expression level was used as cutoff. Low expression of JPX in 116 patients was defined as a value below the 50th percentile, and high above the 50th percentile. *P* values were acquired by Pearson’s chi-square test. **P < 0.01, ***P < 0.001

### JPX promoted lung cancer cell proliferation in vitro and facilitated lung tumor growth in vivo

**Fig. 2:**
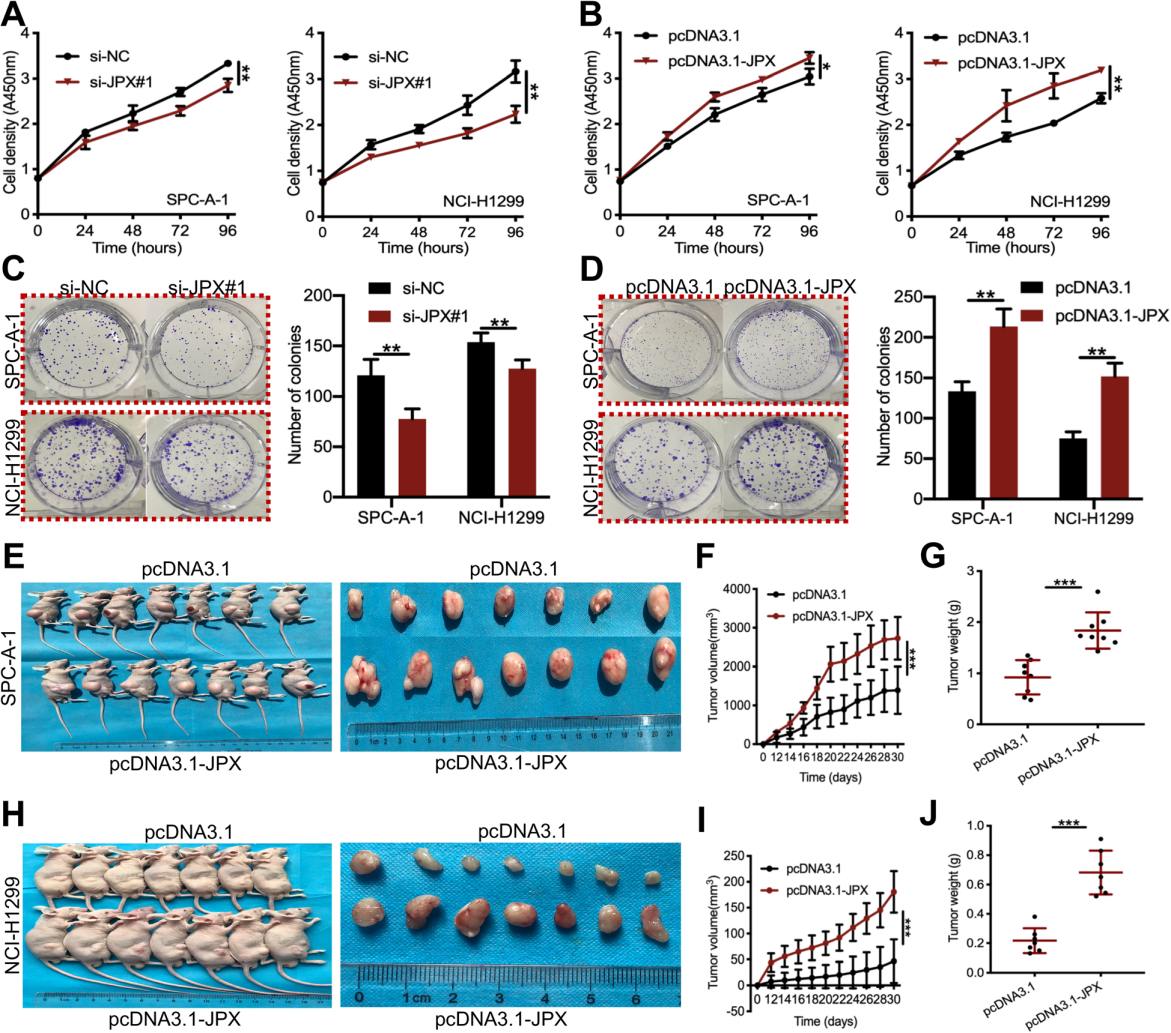
JPX overexpression promoted cell growth and proliferation in vitro and in vivo. The CCK-8 assays were performed to determine the viability of lung cancer cells treated with si-JPX#1 (**a**) or pcDNA3.1-JPX (**b**) in SPC-A1 and NCI-H1299 cells. Colony formation assays were used to detect the proliferation of lung cancer cells after transfection with si-JPX#1 (**c**) or pcDNA3.1-JPX (**d**) in SPC-A1 and NCI-H1299 cells. The bar charts represent the numbers of cell colonies. (**e**) NCI-H1299 cells with empty vector were injected into the upper nude mice (*n* = 7), while NCI-H1299 cells stably expressing JPX were injected into the lower nude mice (n = 7). Representative images of xenograft tumors are shown in the right panel. (**f, g**) Tumor volume and weight in the xenograft mice from the JPX overexpression group and the control group. Data are shown as the mean ± SD in three independent experiments. *P < 0.05, **P < 0.01, ***P < 0.001

To explore the biological function of JPX in lung cancer cells, small interfering RNAs (siRNAs) were used to specifically knockdown JPX expression, whereas the full-length recombinant plasmid with JPX was used to increase JPX expression. By transfecting three JPX siRNAs, we found that si-JPX#1 could significantly downregulate JPX expression by up to 57–71% (Additional file 2, Figure S1A). JPX could be upregulated 21–78 fold when transfected with the JPX recombinant plasmid (Additional file 2, Figure S1B). CCK-8 and colony formation assays revealed that depletion of JPX inhibited the growth and proliferation of SPC-A-1 and NCI-H1299 cells (Fig. 2a and c), while JPX overexpression promoted cell growth and proliferation in both cell lines (Fig. 2b and d). To further investigate the effect of JPX on lung tumor growth in vivo, the xenograft mouse model was generated by subcutaneous injection of lung cancer cell lines (SPC-A-1 and NCI-H1299) stably expressing JPX. With SPC-A-1 cells, the tumor volume and weight in JPX-overexpressing mice were significantly larger and heavier than those in the control mice (Fig. 2e-g). Similar results were also found with NCI-H1299 cells (Fig. 2h-j). Overall, these results indicated that JPX overexpression promoted the lung cancer cell growth and proliferation in vitro and facilitated the lung tumor growth in xenograft mouse model.

### JPX promoted migration and invasion of lung cancer cells

**Fig. 3:**
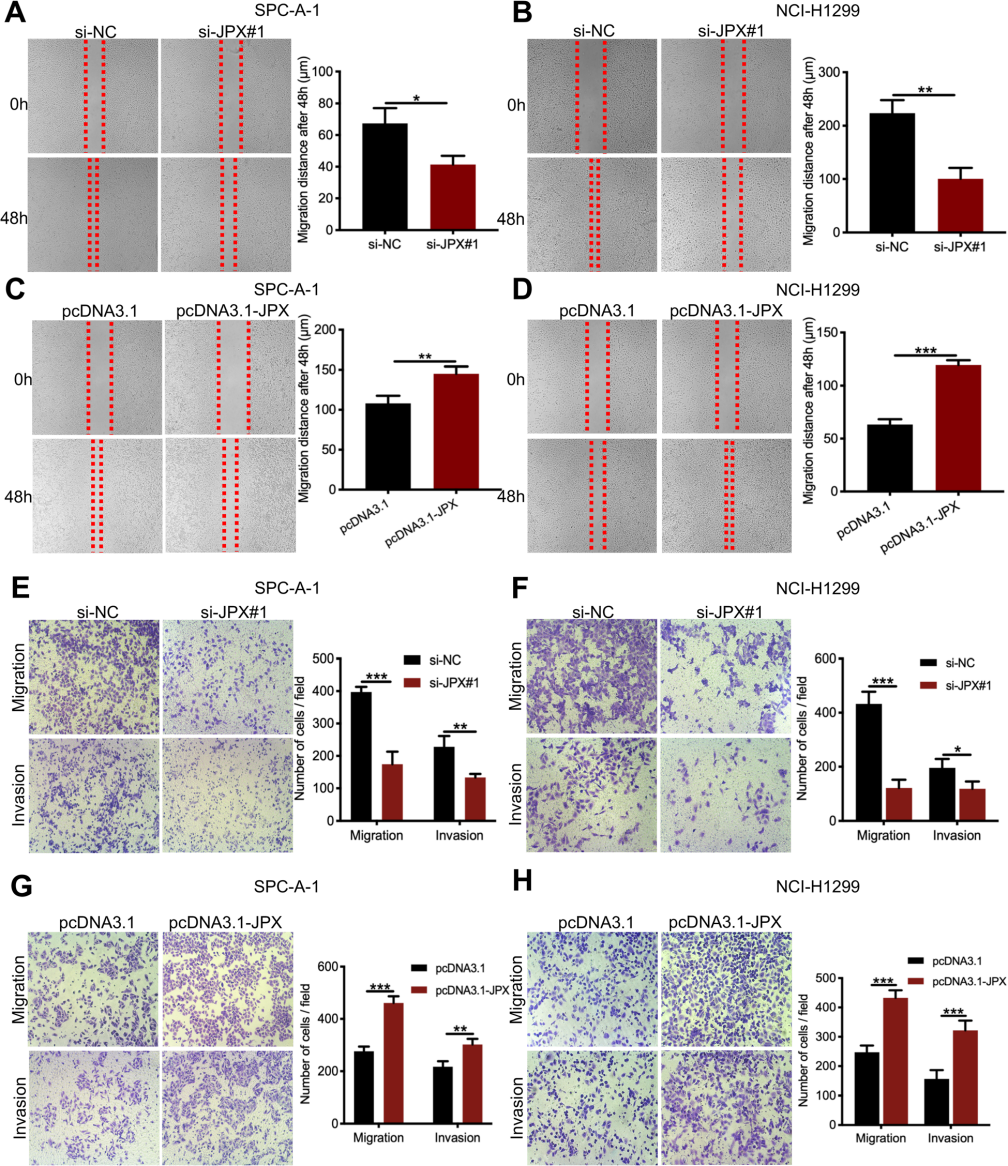
JPX reinforced the migration and invasion of lung cancer cells in vitro. The migration ability of SPC-A-1 and NCI-H1299 cells treated with si-JPX#1 (**a, b**) or pcDNA3.1-JPX (**c, d**) was assessed by wound healing assays. The bar charts represent the distance of the cell migration. The invasion ability of SPC-A-1 and NCI-H1299 cells treated with si-JPX#1 (**e, f**) or pcDNA3.1-JPX (**g, h**) was evaluated by the transwell assays. The bar charts indicate the number of invaded cells. Data are presented as the mean ± SD, *n* = 3. *P < 0.05, **P < 0.01, ***P < 0.001

To determine the effect of JPX on the migration and invasion of lung cancer cells, the wound healing and transwell assays were performed in SPC-A-1 and NCI-H1299 cells. The wound healing assay results showed that knockdown of JPX significantly reduced the migration distance in SPC-A-1 (Fig. 3a) and in NCI-H1299 (Fig. 3b) cells. In contrast, JPX overexpression significantly increased the migration distance in both two cell lines (Fig. 3c and d). The results of migration and invasion assays showed that knockdown of JPX significantly reduced the number of migrated cells in SPC-A-1 (Fig. 3e) and NCI-H1299 (Fig. 3f) cells. Contrarily, JPX overexpression increased the number of migrated cells in SPC-A-1 (Fig. 3g) and NCI-H1299 (Fig. 3h) cells. Thus, the data showed that JPX could enhance the migration and invasion of lung cancer cells in vitro.

### JPX acted as a sponge for miR-33a-5p

**Fig. 4:**
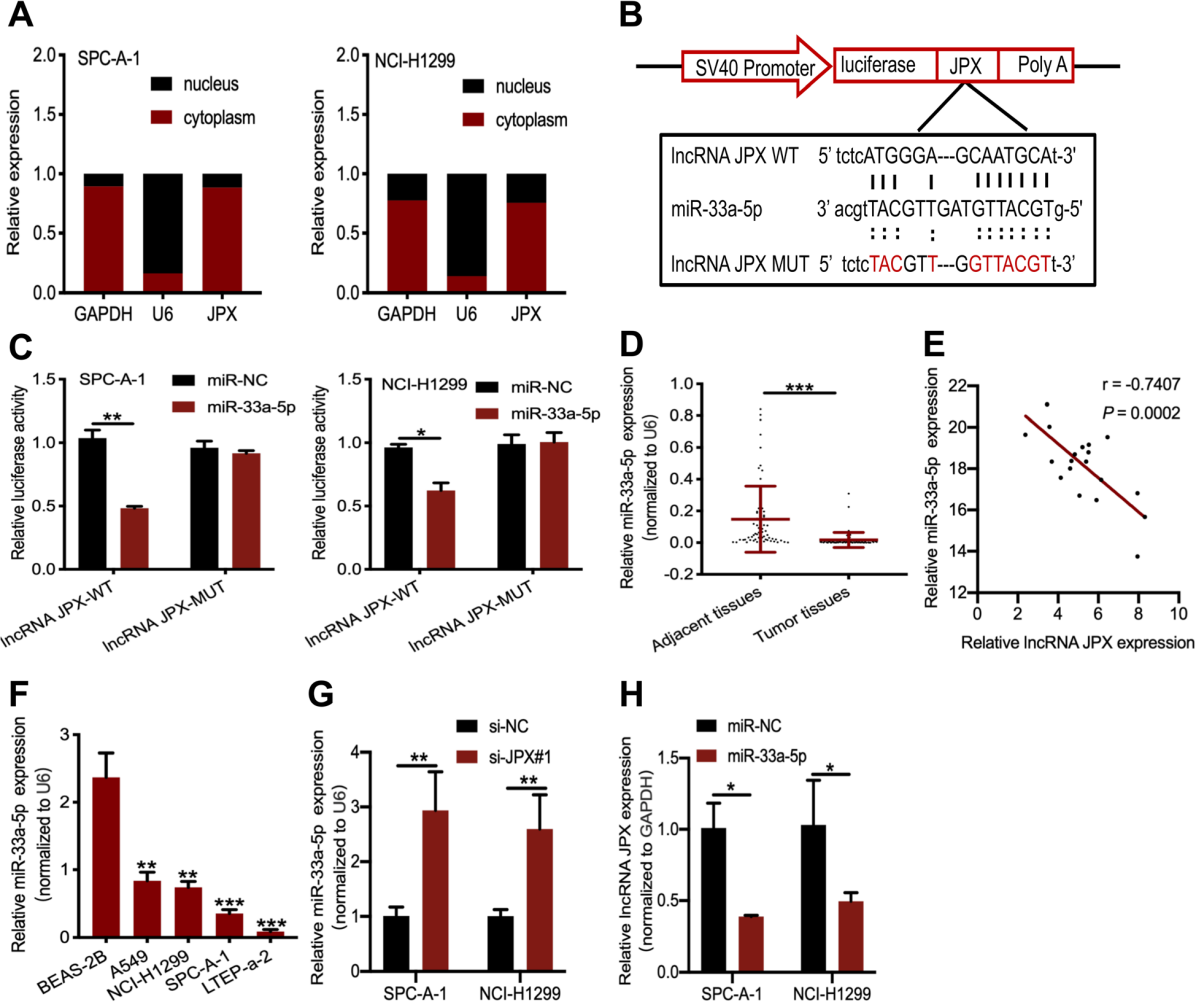
JPX targeted the miR-33a-5p in lung cancer cells as a ceRNA. (**a**) The subcellular position of JPX in the cytoplasm or nucleus. GAPDH and U6 were used as the cytoplasmic and nuclear control, respectively. (**b**)The potential binding sites of miR-33a-5p and JPX. (**c**) Relative luciferase activities of wild type (WT) and mutated (MUT) JPX reporter plasmid in SPC-A-1 and NCI-H1299 cells co-transfected with miR-33a-5p mimics. (**d**) The RT-qPCR assays were utilized to measure miR-33a-5p expression in 65 lung cancer tissues and paired normal tissues. (**e**) Pearson’s correlation analysis determined the relationship between JPX and miR-33a-5p expression in 20 lung cancer tissues. (**f**) Relative expression of miR-33a-5p in lung cancer cell lines compared with that in the normal epithelial cell line. (**g**) Relative miR-33a-5p expression after JPX knockdown. (**h**) Detection of JPX expression by RT-qPCR after overexpression of miR-33a-5p. Data are shown as the mean ± SD based on three independent experiments. *P < 0.05, **P < 0.01, ***P < 0.001

To further confirm the interaction between JPX and miR-33a-5p, we firstly identified the subcellular location of JPX in lung cancer cells. Nuclear-cytoplasmic fractionation showed that JPX was mainly located in the cytoplasm of lung cancer cells (Fig. 4a). The most commonly accepted mechanism of cytoplasmic lncRNAs is that of ceRNAs, which inhibit the regulation of target genes by sponging various miRNAs [24]. To validate the above theory, we subcloned the wild-type (JPX-WT) and mutated (JPX-MUT) miR-33a-5p binding sites into dual-luciferase reporters (Fig. 4b). The luciferase assay showed that transfection of miR-33a-5p mimics significantly reduced the relative luciferase activity of JPX-WT-treated lung cancer cells, but did not affect that of JPX-MUT-treated lung cancer cells (Fig. 4c). To determine the relationship between JPX and miR-33a-5p, we used RT-qPCR assay to evaluate miR-33a-5p expression in lung cancer patients. Compared with the adjacent normal tissues, miR-33a-5p was notably downregulated in lung cancer tissues (Fig. 4d). Interestingly, Pearson correlation analysis showed a negative correlation between the expression level of JPX and miR-33a-5p in lung cancer tissues (Fig. 4e). In contrast to JPX, tiR-33a-5p expression in four lung cancer cell lines was much lower than that in normal lung bronchial epithelial cells (Fig. 4f). In addition, miR-33a-5p was upregulated when the lung cancer cells were transfected with JPX siRNA s (Fig. 4g). However, JPX was downregulated when the lung cancer cells were transfected with miR-33a-5p mimics (Fig. 4h). Taken together, the data suggested that JPX acted as a sponge for miR-33a-5p in lung cancer cells.

### JPX promoted cell proliferation, migration, and invasion of lung cancer cells by regulating miR-33a-5p

**Fig. 5:**
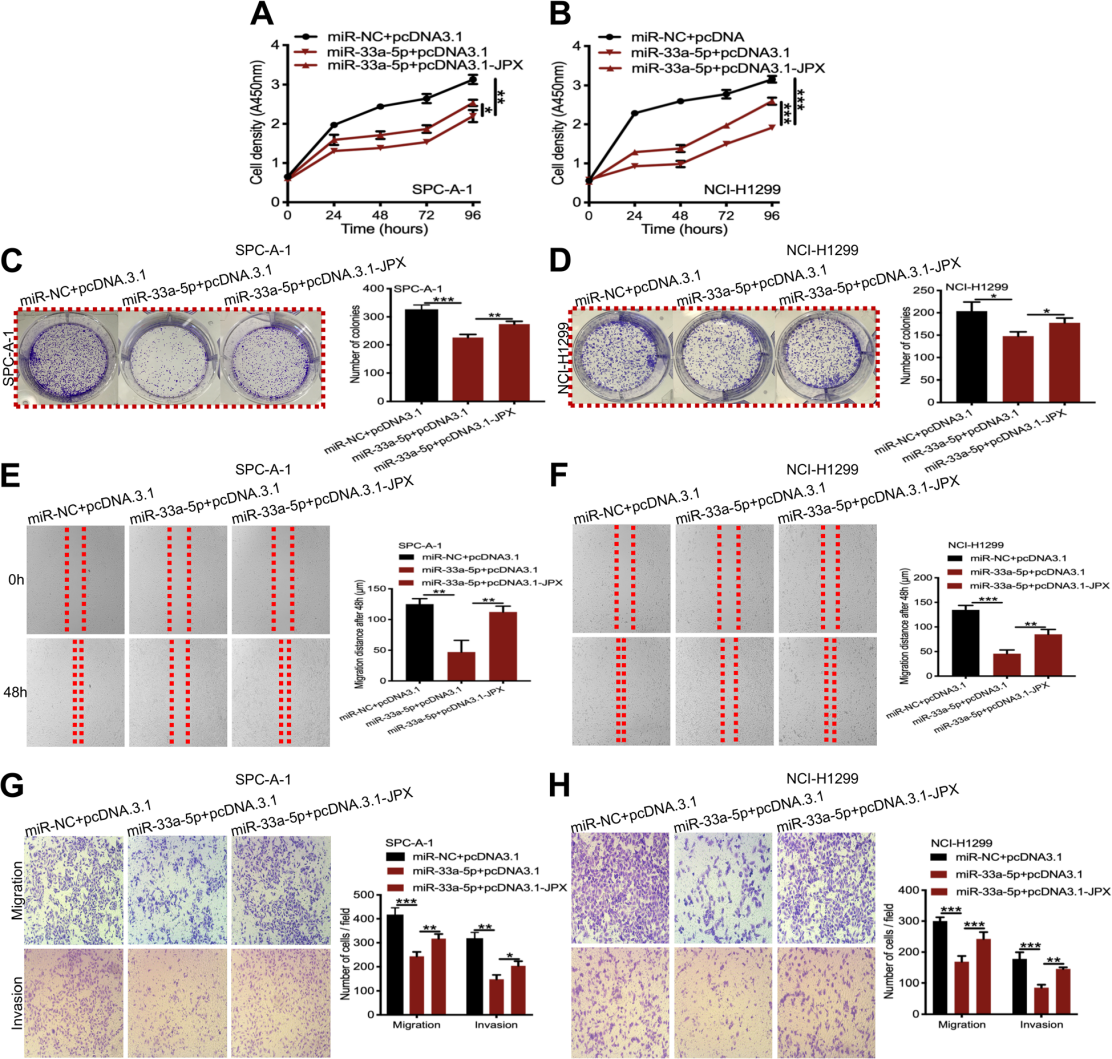
JPX promoted proliferation and metastasis of lung cancer cells by inhibiting miR-33a-5p. (**a, b**) Rescue effects of JPX overexpression on miR-33a-5p mimics-mediated inhibition of cell growth in SPP-A1 and NCI-H1299 cells determined by CCK-8 assays. (**c, d**) Rescue effects of JPX overexpression on miR-33a-5p mimics-mediated inhibition of cell proliferation in SPP-A1 and NCI-H1299 cells determined by colony formation assays. (**e, f**) Rescue effects of JPX overexpression on miR-33a-5p mimics-mediated inhibition of cell migration in SPP-A1 and NCI-H1299 cells determined by wound healing assays. (**g, h**) Rescue effects of JPX overexpression on miR-33a-5p mimics-mediated inhibition of cell invasion in SPP-A1 and NCI-H1299 cells determined by transwell assays. Data are shown as the mean ± SD, *n* = 3. *P < 0.05, **P < 0.01, ***P < 0.001

To investigate whether JPX regulates the cell phenotype via sponging miR-33a-5p, the rescue experiments were employed to detect the effect of JPX and miR-33a-5p on lung cancer cell proliferation, migration, and invasion. The results showed that the cell growth suppression induced by miR-33a-5p overexpression was relieved by restoring JPX in SPC-A-1 (Fig. 5a) and NCI-H1299 (Fig. 5b) cells. In addition, the decrease in cell colony number due to miR-33a-5p overexpression was restored by JPX treatment in the two lung cancer cell lines (Fig. 5c and d). The wound healing assay revealed that miR-33a-5p markedly inhibited cell migration in both SPC-A-1 and NCI-H1299 cells, while the effects were abolished by JPX overexpression (Fig. 5e and f). Similarly, the transwell assay showed that miR-33a-5p significantly suppressed the invasion of SPC-A-1 and NCI-H1299 cells; however, these effects were abrogated by JPX overexpression (Fig. 5g and h). These results indicated that JPX promoted the cell proliferation, migration, and invasion by regulating miR-33a-5p in lung cancer cells.

### JPX promoted lung tumor growth and metastasis in vivo by regulating miR-33a-5p

**Fig. 6:**
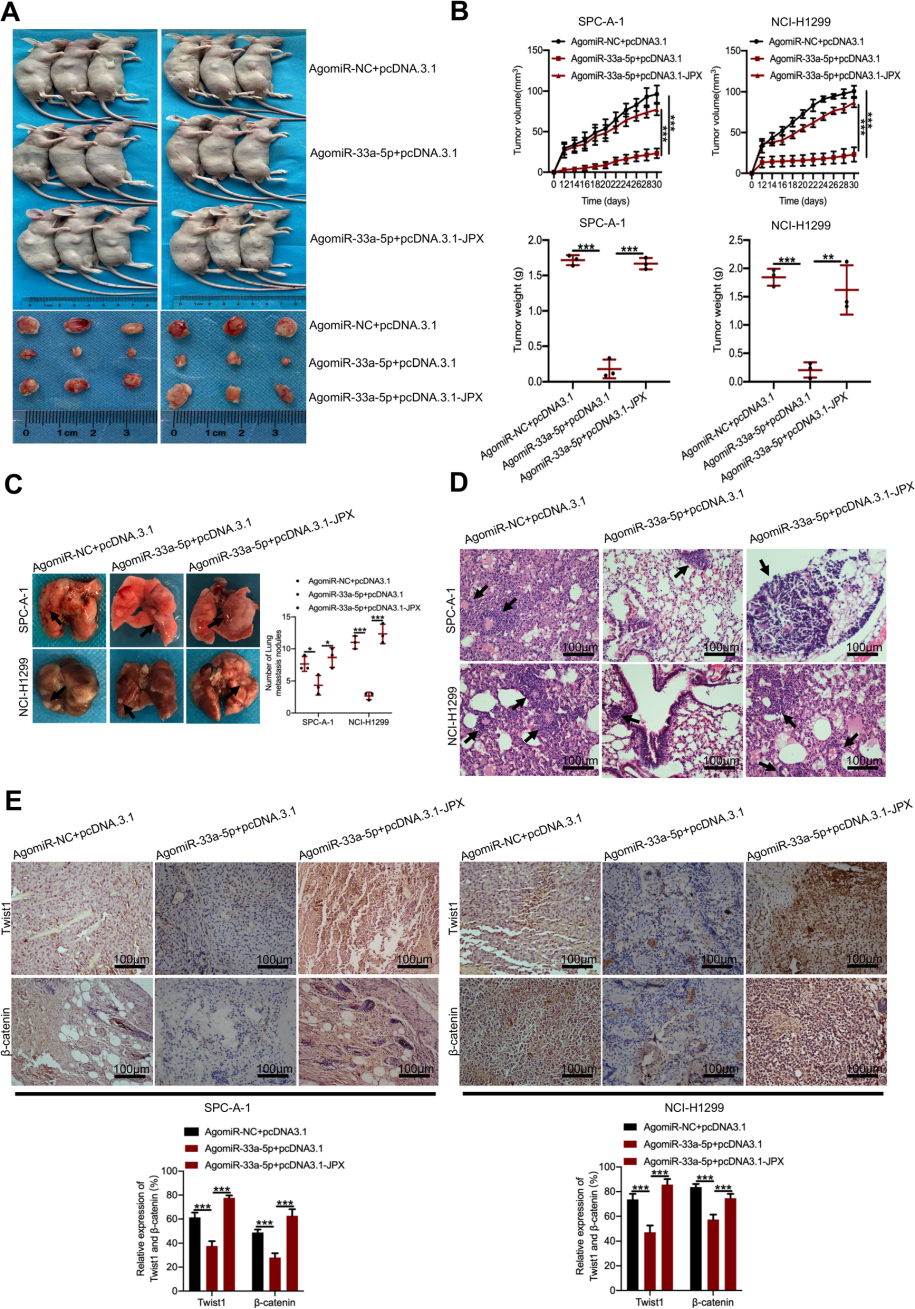
JPX promoted lung tumor growth and metastasis in vivo by regulating miR-33a-5p. Rescue effects of JPX overexpression on AgomiR-33a-5p-mediated inhibition of tumor growth in mice subcutaneously implanted inhibition with SPP-A1 and NCI-H1299 cells. (**a**) Representative subcutaneous xenograft tumors were shown (n = 3). (**b**) Tumor volume and weight were measured. (**c**) Representative images of gross lung tissue lesions and (**d**) H&E-stained sections from the nude mice with tail vein injection in the rescue experiments. (**e**) Representative IHC staining of Twist1 and β-catenin in the tumors from nude mice with subcutaneous implantation in the rescue experiments. Scale bar = 100 μm. Data are presented as the mean ± SD, *n* = 3. *P < 0.05, **P < 0.01, ***P < 0.001

In order to verify whether miR-33a-5p could inhibit lung cancer cell growth and metastasis in vivo and whether JPX could reverse miR-33a-5p-induced suppression of the malignant process of lung cancer cells, co-expression of miR-33a-5p and JPX in nude mice was conducted by intravenous injection. Subcutaneous tumor formation experiments revealed that JPX overexpression significantly promoted the formation of subcutaneous tumors of lung cancer cells, while miR-33a-5p overexpression abrogated the JPX-enhanced tumorigenicity in vivo (Fig. 6a and b). By observing lung tissue surfaces in the nude mice metastasis model, it was found that the miR-33a-5p upregulation group could reduce the metastatic lesion compared to the normal controls, while JPX overexpression restored these effects (Fig. 6c). H&E staining showed that miR-33a-5p markedly decreased the number of metastatic lung nodules, whereas the effects were abolished in the JPX overexpression group (Fig. 6d). In addition, IHC assay showed that both Twist1 and β-catenin were significantly increased by upregulating JPX; these effects could be reversed by increasing miR-33a-5p levels (Fig. 6e). These results indicated that JPX promoted lung tumor growth and metastasis in vivo by regulating miR-33a-5p.

### JPX and *Twist1* were coordinately upregulated in lung cancer tissues and cells

**Fig. 7:**
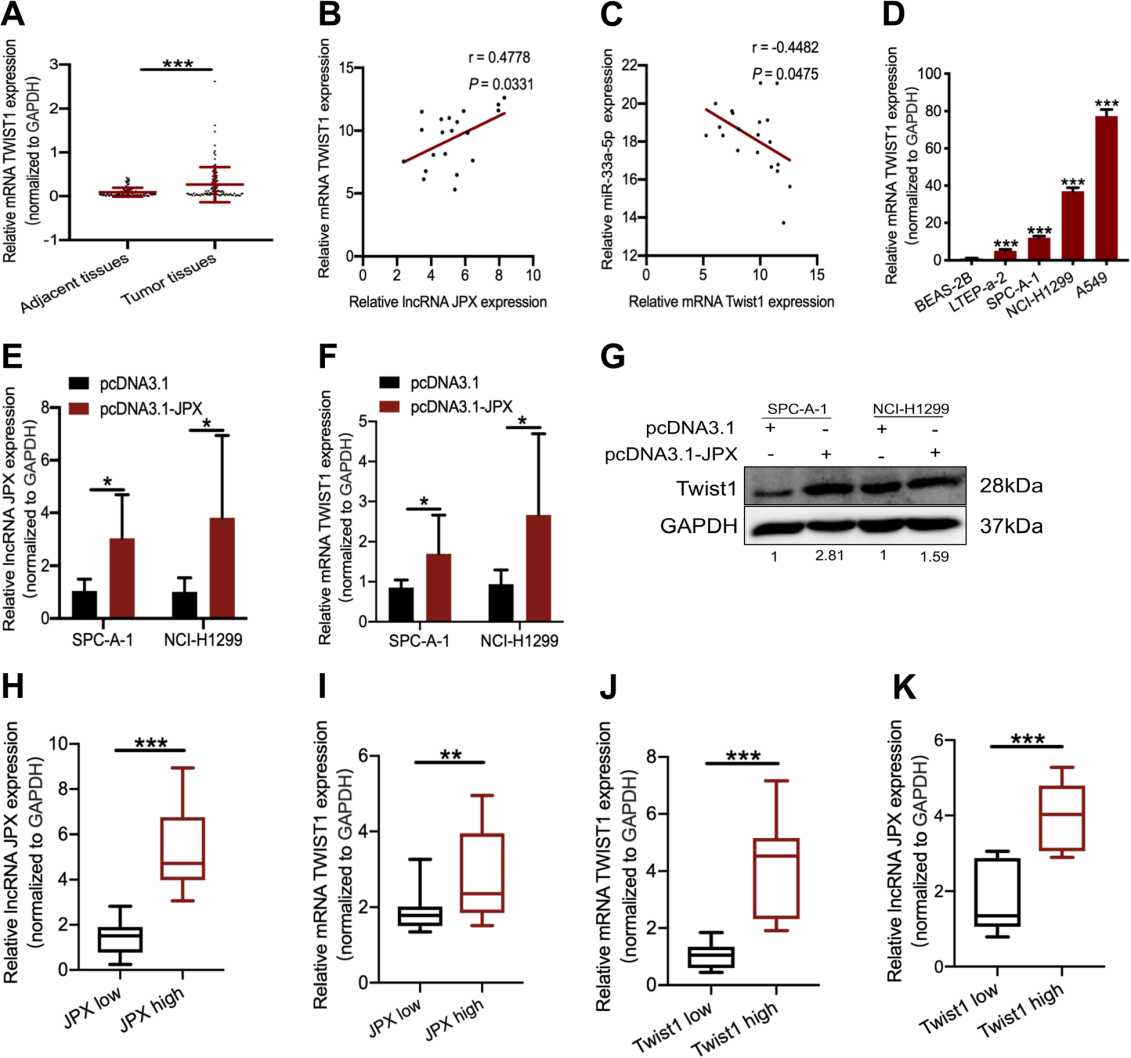
JPX promoted *Twist1* expression in lung cancer cells. (**a**) Relative Twist1 expression in 95 lung cancer tissues and matched noncancerous tissues. (**b**) The relationship between *Twist1* and JPX expression in 20 lung cancer tissues using Pearson’s correlation analysis. (**c**) Correlation analysis between JPX and miR-33a-5p expression in 20 lung cancer tissues. (**d**) Relative *Twist1* expression in lung cancer cell lines compared with that in the normal epithelial cell line. The relative expression levels of JPX (**e**) and Twist1 (**f**) in tumor tissues from the xenograft models. (**g**) Twist1 protein expression when transfected with enhanced JPX. (**h, i**) Based on JPX expression, the qRT-PCR data from clinical samples was classified as JPX high and JPX low. Relative Twist1 mRNA expression was compared with JPX and using box plots. (**j, k**) Based on Twist1 mRNA expression, the qRT-PCR data from clinical samples were classified as the “Twist1 high” and the “Twist1 low”. Relative JPX expression was compared with Twist1 mRNA using box plots. Data are shown as the mean ± SD, n = 3. *P < 0.05, **P < 0.01, ***P < 0.001

Since studies have shown that miR-33a-5p negatively regulates its target gene, *Twist1* [16, 25], we aimed to further investigate the relationship between JPX and *Twist1* in lung cancer. *Twist1* expression was verified in 95 pairs of lung cancer tissues and adjacent tissues. It was found that *Twist1* was highly expressed in lung cancer tissues compared to that in adjacent tissues (Fig. 7a). Pearson’s correlation analysis showed that the JPX expression was positively correlated with *Twist1* in lung cancer patients (Fig. 7b). As expected, miR-33a-5p was negatively correlated with *Twist1* in lung cancer patients (Fig. 7c). *Twist1* was highly expressed in lung cancer cells, which was consistent with JPX expression (Fig. 7d). In the xenograft tumor mice, RT-qPCR showed that JPX expression was remarkably upregulated in tumor tissues (Fig. 7e). Moreover, JPX overexpression induced an increase in *Twist1* expression in tumor tissues at the RNA (Fig. 7f) and protein (Fig. 7g) levels. To further assess the relevance of JPX and *Twist1*, we divided the lung cancerous specimens into two groups: the “JPX high” and “JPX low” groups, which had higher or lower JPX expression, respectively, in tumor tissues than that in the paired adjacent non-tumor tissues (Fig. 7h). Notably, the mRNA levels of *Twist1* were higher in the “JPX high” group than in the “JPX low” group (Fig. 7i). Similarly, two groups named the “*Twist1* mRNA high” and the “*Twist1* mRNA low” were generated to distinguish the relative expression of *Twist1* mRNAs (Fig. 7j). As expected, JPX RNA levels were obviously higher in the “*Twist1* mRNA high” subset than in the “*Twist1* mRNA low” subset (Fig. 7k). Collectively, the data indicated that JPX and *Twist1* were coordinately upregulated in lung cancer.

### JPX/miR-33a-5p/Twist1 axis participated in lung cancer cell EMT progression via the Wnt/β-catenin signaling

**Fig. 8:**
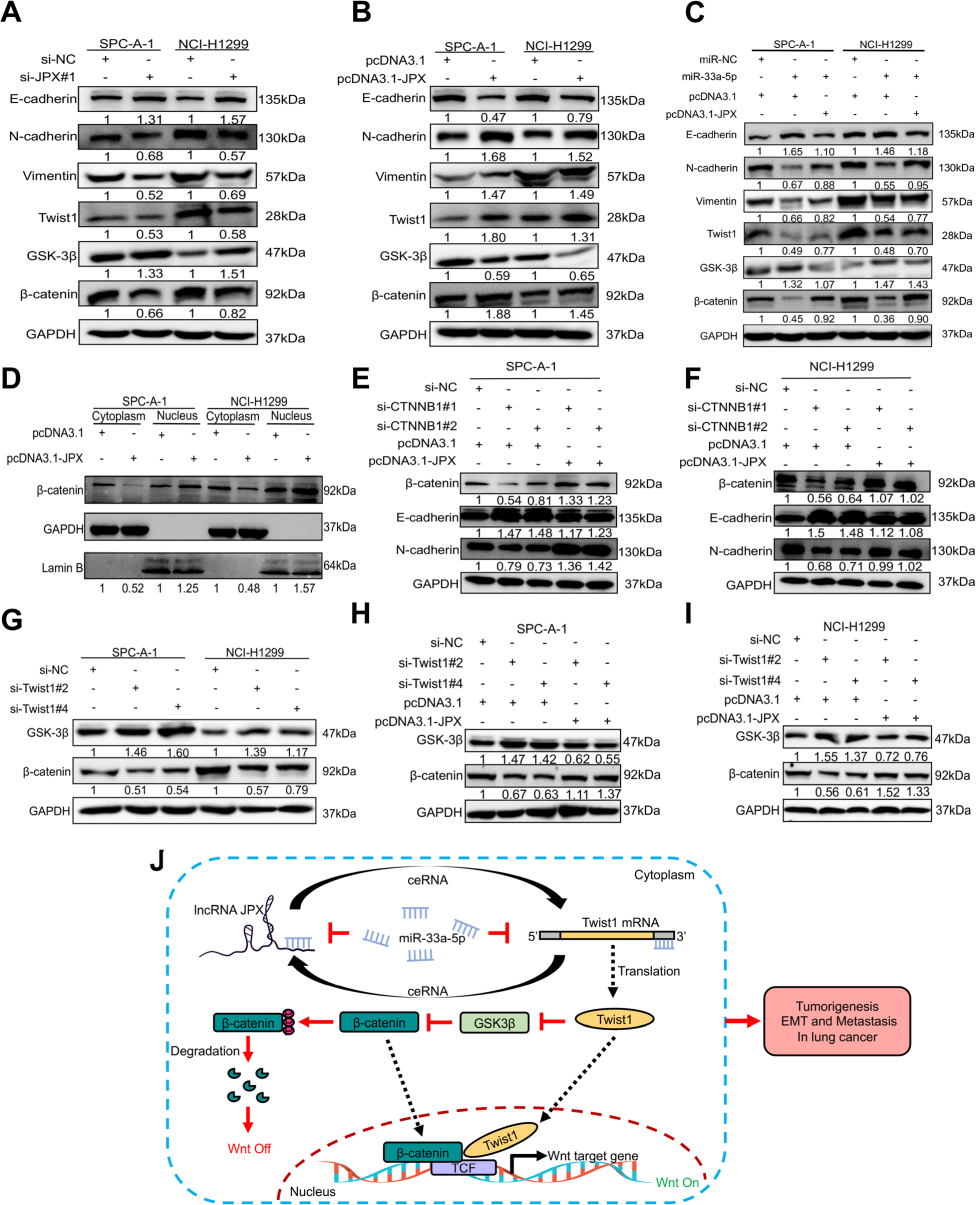
JPX/miR-33a-5p/Twist1 promoted EMT progression in lung cancer cells by activating the Wnt/β-catenin pathway. Immunoblot assay of E-cadherin, N-cadherin, Vimentin, Twist1, GSK-3β and β-catenin proteins in SPC-A-1 and NCI-H1299 cells transfected with si-JPX#1 (**a**), pcDNA3.1-JPX (**b**), and miR-33a-5p + pcDNA3.1-JPX (**c**). Western blot analysis for β-catenin, E-cadherin, and N-cadherin in SPC-A-1 and NCI-H1299 cells after transfection with pcDNA3.1-JPX (**d**), and si-CTNNB1+ pcDNA3.1-JPX (**e, f**). Western blotting was conducted to verify the protein expression of GSK-3β and β-catenin in SPC-A-1 and NCI-H1299 cells transfected with si-Twist1#2/#4 (**g**) and si-Twist1#2/#4 + pcDNA3.1-JPX (**h, i**). The numbers indicate the quantification of relative protein amount. GAPDH was used as an internal control. (**j**) Schematic diagram of the regulatory mechanism of the JPX/miR-33a-5p/Twist1 axis in promoting lung cancer cells proliferation and metastasis. Twist1 could participate in the EMT process through the Wnt/β-catenin pathway, ultimately affecting the proliferation and metastasis of lung cancer cells, which could be inhibited by miR-33a-5p and enhanced by JPX as a ceRNA. Data are shown as the mean ± SD based on three independent experiments

Wnt/β-catenin signaling, a driver of EMT, was investigated by immunological blotting to discern the molecular mechanism underlying the JPX/miR-33a-5p/Twist1 axis in EMT progression. The results showed that the epithelial biomarker of E-cadherin, and GSK-3β, a key regulator of in Wnt/β-catenin signal pathway, were upregulated; whereas the mesenchymal biomarkers of N-cadherin and Vimentin, as well as Twist1 as an inducer of EMT, and β-catenin were downregulated in lung cancer cells with JPX knockdown (Fig. 8a). In contrast, JPX overexpression resulted in the opposite changes in the EMT biomarkers and key regulators of the Wnt/β-catenin pathway (Fig. 8b). To reveal whether miR-33a-5p participated in the EMT process by regulating Wnt/β-catenin signaling, we transfected miR-33a-5p mimics into SPC-A-1 and NCI-H1299 cells and found that the protein expression levels of E-cadherin and GSK-3β were increased, whereas those of N-cadherin, Vimentin, Twist1, and β-catenin were decreased. Importantly, JPX overexpression could restore the miR-33a-5p-induced abnormal expression of EMT- and Wnt/β-catenin pathway-associated proteins in lung cancer cells (Fig. 8c). The cytosolic protein β-catenin is a molecular switch of the canonical Wnt signaling pathway, and it accumulates in the cytoplasm to activate the transcription of a series of Wnt signaling target genes [26]. Our results demonstrated that JPX could positively regulate β-catenin protein expression, so we speculated that JPX could promote β-catenin to accumulate in the cytoplasm and then enter the nucleus, thereby activating the Wnt signaling pathway. To verify our hypothesis, we isolated nuclear and cytoplasmic proteins in lung cancer cell lines (SPC-A-1 and NCI-H1299) stably expressing JPX and their controls and found that β-catenin expression was reduced in the cytoplasm and significantly increased in the nucleus (Fig. 8d). The results indicated that JPX could promote the transfer of β-catenin from the cytoplasm to the nucleus to activate the transcription of target genes. In addition, to further prove that JPX could upregulate β-catenin expression, we transfected siRNAs of CTNNB1, which is a gene encoding β-catenin, in lung cancer cells (Additional file 2, Figure S2A and B). It was found that si-CTNNB1 could reduce the expression levels of β-catenin and N-cadherin but increase that of E-cadherin (Fig. 8e). JPX overexpression could restore the CTNNB1 knock down-induced abnormal expression of β-catenin and EMT-related markers (Fig. 8f). To further confirm whether Twist1 is directly involved in the Wnt/β-catenin pathway, we first screened four synthesized siRNAs and found that two of them (named si-Twist1#2, si-Twist1#4) significantly reduced the expression of Twist1 in two lung cancer cell lines (Additional file 2, Figure S3A and B). Next, it was found that Twist1 knockdown led to an increase in GSK-3β and a decrease in β-catenin in both SPC-A-1 and NCI-H1299 cells (Fig. 8g). Interestingly, JPX overexpression could decrease GSK-3β expression and increase β-catenin expression upon the siRNA-mediated Twist1 knockdown in SPC-A-1 (Fig. 8h) and NCI-H1299 (Fig. 8i) cells.

Together, the data demonstrated that JPX regulated miR-33a-5p/Twist1-mediated EMT progression by activating the Wnt/β-catenin signaling (Fig. 8j).

## Discussion

Although encouraging progress has been made in understanding the molecular mechanisms of lung cancer development, the prognosis of patients with advanced lung cancer remains unfavorable [27]. Recent studies have shown that abnormally expressed lncRNAs are closely related to lung cancer occurrence and development [28–30]. Specifically, lncRNA HCP5 was found to be upregulated in lung adenocarcinoma (LUAD), resulting in increases of *Snail* and *Slug* to promote EMT progression by adsorbing miR-203 [31]. As an early specific antisense lncRNA, SBF2-AS1 promoted LUAD tumorigenesis through substantially decreased miR-338-3p and miR-362-3p and substantially increased *E2F1*, and served as a prognostic marker and potential therapeutic target for LUAD [32]. Based on our previous work, we have found that miR-33a-5p negatively regulated the target gene of *Twist1* and participated in the EMT process of lung cancer cells. We combined an lncRNA microarray and bioinformatic prediction to screen out lncRNA JPX, which has potential binding sites with miR-33a-5p and is associated with lung cancer tumorigenesis. In this study, we showed that JPX was significantly upregulated in lung cancer tissues and cells. Importantly, JPX promoted lung cancer malignant processes and tumor growth in vivo. The results indicate that JPX plays an oncogenic role in lung cancer.

JPX is a molecular switch that inactivates the X chromosome [33, 34]. Recent study has shown that exosomal JPX from hepatocellular carcinoma (HCC) cells promotes XIST expression by inhibiting the function of CCCTC-binding factor (CTCF) in blood cells [35]. Relevant research shows that human JPX and its mouse homolog of lncRNA Jpx have great differences in their nucleotide sequences and RNA secondary structures, but both lncRNAs show strong binding to CTCF, and human JPX can functionally compensate for the loss of Jpx in mouse embryonic stem cells [36]. It has also been shown that JPX is lowly expressed in HCC and inhibits HepG2 cell growth or tumorigenesis in a XIST-dependent manner, revealing that JPX has a tumor-suppressing effect in HCC [37]. In addition, highly expressed JPX shows poor prognosis; promotes the proliferation, invasion, and migration of human ovarian cancer cells, and inhibits cell apoptosis by activating the PI3K/Akt/mTOR signaling [38]. Recently, there have been reports on JPX in lung cancer. As an oncogene, JPX is significantly upregulated in NSCLC tissues and is associated with poor prognosis; JPX upregulates cyclin D2 expression in the ceRNA mechanism by interacting with miR-145-5p, which stimulates NSCLC development and progression [39]. These studies show that JPX plays different roles in different types of human cancers. On the one hand, JPX acts as an oncogene to promote the development of ovarian and lung cancer. On the other hand, JPX acts as a tumor suppressor gene to inhibit HCC development. In our study, we found for the first time that JPX was highly expressed in lung cancer patients and was significantly linked to tumor size and TNM stage. Interestingly, JPX expression was higher in patients with advanced lung cancer than in those with early lung cancer. Consistently, JPX expression was also higher in patients with metastatic lung cancer than in those without metastasis. We further revealed a negative correlation between JPX and miR-33a-5p in lung cancer patients. The miR-33a-5p-induced inhibition of cell growth and metastasis could be restored by JPX overexpression. Additionally, Twist1*,*a target of miR-33a-5p, was found to be coordinately upregulated with JPX in lung cancer. These findings indicate that JPX plays an oncogenic role via its interaction with miR-33a-5p and Twist1.

In the past decade, ceRNAs have become a very important class of post-transcriptional regulators that affect tumor occurrence and development by altering the corresponding gene expression through miRNA-mediated mechanism [40]. As a type of ceRNA, lncRNAs can act as molecular sponges to adsorb miRNAs through the same miRNA response elements (MREs), thereby regulating their target genes and ultimately affecting tumor progression. For instance, lncRNA CA7–4 regulated the autophagy and apoptosis of vascular endothelial cells by inducing miR-877-3p and miR-5680 under high-glucose condition [41]. Similarly, LINC01234 acted as a ceRNA of miR-642a-5p, resulting in the suppression of the endogenous serine hydroxymethyl transferase 2 (SHMT2), suggesting that the LINC01234-miR642a-5p-SHMT2 axis plays a key role in colon cancer [42]. There are also other lncRNAs that play crucial roles as ceRNAs in cancers development [43, 44]. The present work demonstrated that lncRNA JPX, miRNA-33a-5p, and Twist1 constituted a ceRNA network to regulate lung cancer growth and metastasis. However, the ceRNA hypothesis is still in the verification stage, and much research is needed to identify the abundance of the three components and verify the functional activities of ceRNAs.

It has been reported that EMT and the associated Wnt/β-catenin pathway can be important drivers of tumor growth and metastasis [45]. Twist1 is a member of the basic helix-loop-helix transcription factor family and is an important transcription factor that induces EMT, migration and invasion in cancer cells [18, 19]. Furthermore, Twist1 is highly expressed and acts as an oncogene in many invasive types of cancers, such as lung cancer [46], breast cancer [47], and HCC [48]. Additionally, studies have shown that Twist1 affects the cancerous behavior of tumor cells via the Wnt/β-catenin pathway [49]. In the current study, we found that JPX could increase Twist1 expression by adsorbing miR-33a-5p, thereby activating Wnt/β-catenin signaling pathway to promote EMT progression in lung cancer cells. However, whether Twist1 participates in the Wnt/β-catenin pathway directly or indirectly, and whether JPX affects Twist1 expression or regulates other pathways through RNA-binding proteins to promote lung cancer development remain unclear. The above speculation needs further investigation. Importantly, recent studies have shown that lncRNA is closely related to the stemness of cancer cells [50]. For example, lncRNA CCAT1 which is notably upregulated in breast cancer stem cells (BCSCs) and contributes to the stemness of BCSCs [51]; lncRNA SPRY4-IT1 increases TCF7L2 expression by targeting miR-6882-3p, thereby promoting breast cancer cell proliferation and stemness as well as BCSC renewal and maintenance [52]; and lncRNA LOXL1-AS1 facilitates the stemness of gastric carcinoma through regulating the miR-708-5p/USF1 axis [53]. Although we have not tested the function of JPX in lung cancer stem cells at present, our results provide a deeper understanding of the role of JPX in lung cancer cells as well as a new direction for future research.

## Conclusions

In summary, our results demonstrate that the lncRNA JPX/miRNA-33a-5p/Twist1 axis may act as a new ceRNA regulatory network, participating in the EMT process by activating the Wnt/β-catenin signaling pathway, thus accelerating the malignant processes of lung cancer. These findings suggest that JPX may serve as a potential therapeutic target and a novel biomarker for the precise treatment of lung cancer.

## Supplementary information

**Additional file 1: Table S1** Primer sequences. **Table S2** RNA oligonucleotide sequences.

**Additional file 2: Figure S1** Knockdown and overexpression of JPX in lung cancer cells. **Figure S2** Knockdown of CTNNB in lung cancer cells. **Figure S3** Knockdown of Twist1 in lung cancer cells.

## Data Availability

The authors declare that all the other data supporting the findings of this study are available within the article and its additional files and from the corresponding author upon reasonable request.

## Abbreviations

ceRNA: Competitive endogenous RNA
CTCF: CCCTC-binding factor
DMEM: Dulbecco’s modified Eagle’s medium
GBC: Gallbladder cancer
HCC: Hepatocellular carcinoma
HK2: Hexokinase 2
lncRNA: Long noncoding RNA
LUAD: Lung adenocarcinoma
miRNA: microRNA
MRE: miRNA response elements
NSCLC: Non-small cell lung cancer
qPCR: Quantitative polymerase chain reaction
RBP: RNA-binding proteins
SHMT2: Serine hydroxymethyl transferase 2
siRNA: Small interfering RNA
