# Supplementary material for: lncRNA JPX/miR-33a-5p/Twist1 axis regulates tumorigenesis and metastasis of lung cancer by activating Wnt/β-catenin signaling
Source: Mol Cancer. 2020 Jan 15;19:9. doi: 10.1186/s12943-020-1133-9 (PMC6961326; doi:10.1186/s12943-020-1133-9)
Supplement: Supplementary file 1 — Additional file 1: Table S1 Primer sequences. Table S2 RNA oligonucleotide sequences. [file 12943_2020_1133_MOESM1_ESM.docx]

**lncRNA JPX/miR-33a-5p/Twist1 axis regulates tumorigenesis and metastasis of lung cancer by activating Wnt/β-catenin signaling**

Jinchang Pan^1,2^, Shuai Fang^1,2^, Haihua Tian^1,2,5^, Chengwei Zhou^3^, Xiaodong Zhao^3^, Hui Tian^4^, Jinxian He^4^, Weiyu Shen^4^, Xiaodan Meng^1,2^, Xiaofeng Jin^1,2^and Zhaohui Gong^1,2*^

**Supplementary Information**

**Table S1** Primer sequences.

**Table S2** RNA oligonucleotide sequences.

**Table S1** Primer sequences.

| **Primer** | **Forward primer (5’ to 3’)** | **Reverse primer (5’ to 3’)** |
| --- | --- | --- |
| JPX (for qPCR) | TGCAGTCAGAAGGGAGCAAT | CACCGTCATCAGGCTGTCTT |
| Twist1 (for qPCR) | GGCTCAGCTACGCCTTCTC | TCCATTTTCTCCTTCTCTGGAA |
| miR-33a-5p (for qPCR) | CCTCATAAGCGGTGCATTGTA | TATGCTTGTTCTCGTCTCTGTGTC |
| GAPDH (for qPCR) | ACCCACTCCTCCACCTTTGAC | TGTTGCTGTAGCCAAATTCGTT |
| CNNB1 (for qPCR) | GTGCAATTCCTGAGCTGACA | CTTAAAGATGGCCAGCAAGC |
| U6 snRNA (for qPCR) | CTCGCTTCGGCAGCACA | AACGCTTCACGAATTTGCGT |
| JPX (for PCR) | AAAGAATTCTCGGAAGACTTAAGATG | AAAGCGGCCGCCATGTTTCTAATTAGC |
| JPX-WT (for PCR) | AAAGCTAGCTCTCATGGGAGCAATGCAT | AAACTCGAGCACATTACATGATTTCCAA |
| JPX-MUT (for PCR) | AAAGCTAGCTCTCTACGTTGGTTACGTT | AAACTCGAGCACATTACATGATTTCCAA |

**Table S2** RNA oligonucleotide sequences.

| **RNA oligos** | **Sequences** |
| --- | --- |
| miRNA-NC | Sense: 5’- UUCUCCGAACGUGUCACGUTT -3’  Anti-sense: 5’- ACGUGACACGUUCGGAGAATT -3’ |
| miR-33a-5p mimics | Sense: 5’- GUGCAUUGUAGUUGCAUUGCA -3’  Anti-sense: 5’- CAAUGCAACUACAAUGACCUU -3’ |
| AgomiR-NC | Sense: 5’- UUCUCCGAACGUGUCACGUTT -3’  Anti-sense: 5’- ACGUGACACGUUCGGAGAATT -3’ |
| AgomiR-33a-5p | Sense: 5’- GUGCAUUGUAGUUGCAUUGCA -3’  Anti-sense: 5’- CAAUGCAACUACAAUGACCUU -3’ |
| siRNA-NC | Sense: 5’- UUCUCCGAACGUGUCACGUTT -3’  Anti-sense: 5’- ACGUGACACGUUCGGAGAATT -3’ |
| si-JPX#1 | Sense: 5’- CCAGUUAAUAGUAUUGUGUTT -3’  Anti-sense: 5’- ACACAAUACUAUUAACUGGTT -3’ |
| si-JPX#2 | Sense: 5’- CGCCUCAGUUUCAGAUUGATT -3’  Anti-sense: 5’- UCAAUCUGAAACUGAGGCGTT -3’ |
| si-JPX#3 | Sense: 5’- GAGCAACCAUAAAGAGUCUTT -3’  Anti-sense: 5’- AGACUCUUUAUGGUUGCUCTT -3’ |
| si-Twist1#1 | Sense: 5’- GCAUCACUAUGGACUUUCUTT -3’  Anti-sense: 5’- AGAAAGUCCAUAGUGAUGCTT -3’ |
| si-Twist1#2 | Sense: 5’- CCUGAGCAACAGCGAGGAATT -3’  Anti-sense: 5’- UUCCUCGCUGUUGCUCAGGTT -3’ |
| si-Twist1#3 | Sense: 5’- GCAAGAUUCAGACCCUCAATT -3’  Anti-sense: 5’- UUGAGGGUCUGAAUCUUGCTT -3’ |
| si-Twist1#4 | Sense: 5’- GAUGGCAAGCUGCAGCUAUTT -3’  Anti-sense: 5’- AUAGCUGCAGCUUGCCAUCTT -3’ |
| si-CTNNB1#1 | Sense: 5’- GGGAGTGGTTTAGGCTATTTG -3’  Anti-sense: 5’- CAAATAGCCTAAACCACTCCC-3 -3’ |
| si-CTNNB1#2 | Sense: 5’- UUGUUAUCAGAGGACUAAAUA -3’  Anti-sense: 5’- UAUUUAGUCCUCUGAUAACAA -3’ |
