# Supplementary material for: lncRNA JPX/miR-33a-5p/Twist1 axis regulates tumorigenesis and metastasis of lung cancer by activating Wnt/β-catenin signaling
Source: Mol Cancer. 2020 Jan 15;19:9. doi: 10.1186/s12943-020-1133-9 (PMC6961326; doi:10.1186/s12943-020-1133-9)
Supplement: Supplementary file 2 — Additional file 2: Figure S1 Knockdown and overexpression of JPX in lung cancer cells. Figure S2 Knockdown of CTNNB in lung cancer cells. Figure S3 Knockdown of Twist1 in lung cancer cells. [file 12943_2020_1133_MOESM2_ESM.docx]

**lncRNA JPX/miR-33a-5p/Twist1 axis regulates tumorigenesis and metastasis of lung cancer by activating Wnt/β-catenin signaling**

Jinchang Pan^1,2^, Shuai Fang^1,2^, Haihua Tian^1,2,5^, Chengwei Zhou^3^, Xiaodong Zhao^3^, Hui Tian^4^, Jinxian He^4^, Weiyu Shen^4^, Xiaodan Meng^1,2^, Xiaofeng Jin^1,2^and Zhaohui Gong^1,2*^

**Supplementary Information**

**Figure S1 Knockdown and overexpression of JPX in lung cancer cells.**

**Figure S2 Knockdown of CTNNB1 in lung cancer cells.**

**Figure S3 Knockdown of Twist1 in lung cancer cells.**


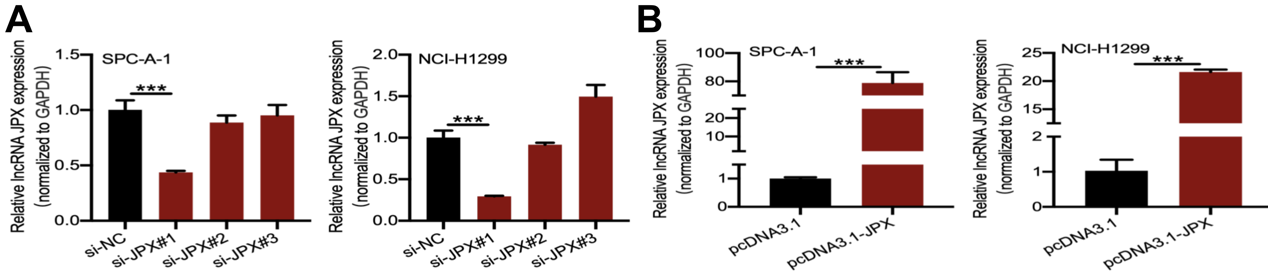


**Figure S1 Knockdown and overexpression of JPX in lung cancer cells. A**) RT-qPCR analysis of relative JPX expression after transfection of JPX siRNA (si-JPX) and the corresponding controls (si-NC) in SPC-A-1 and NCI-H1299 cells. **B**) RT-qPCR analysis of the relative JPX expression after transfection of JPX overexpressing plasmid (pcDNA3.1-JPX) and the corresponding empty plasmid (pcDNA3.1) in SPC-A-1 and NCI-H1299 cells. ****P* < 0.001.


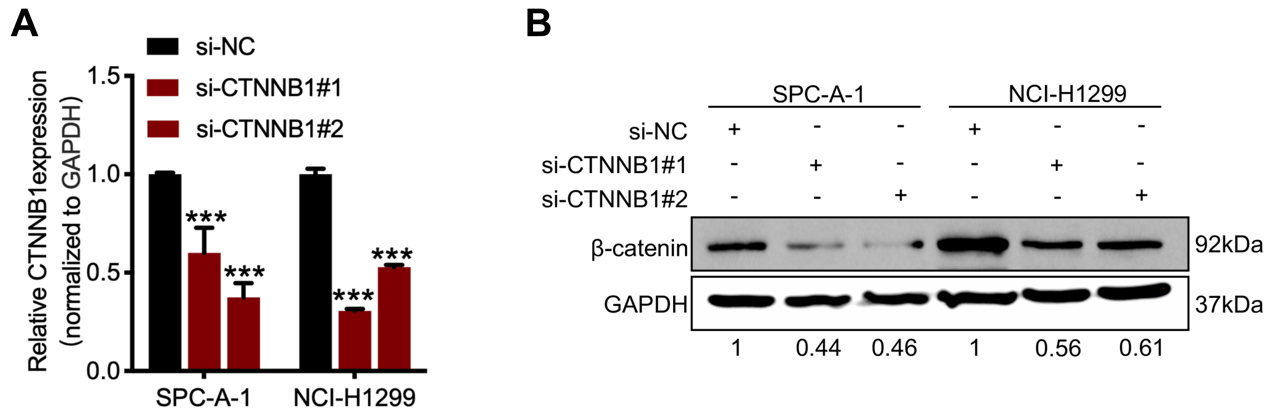


**Figure S2 Knockdown of CTNNB1 in lung cancer cells. A**) RT-qPCR analysis of relative CTNNB1 expression after transfection of CTNNB1 siRNA (si- CTNNB1) and the corresponding controls (si-NC) in SPC-A-1 and NCI-H1299 cells. **B**) Western blot analysis of β-catenin protein in SPC-A1 and NCI-H1299 cells transfected with control siRNA (si-NC) and siRNA to CTNNB1 (si- CTNNB1#1, si- CTNNB1#2).


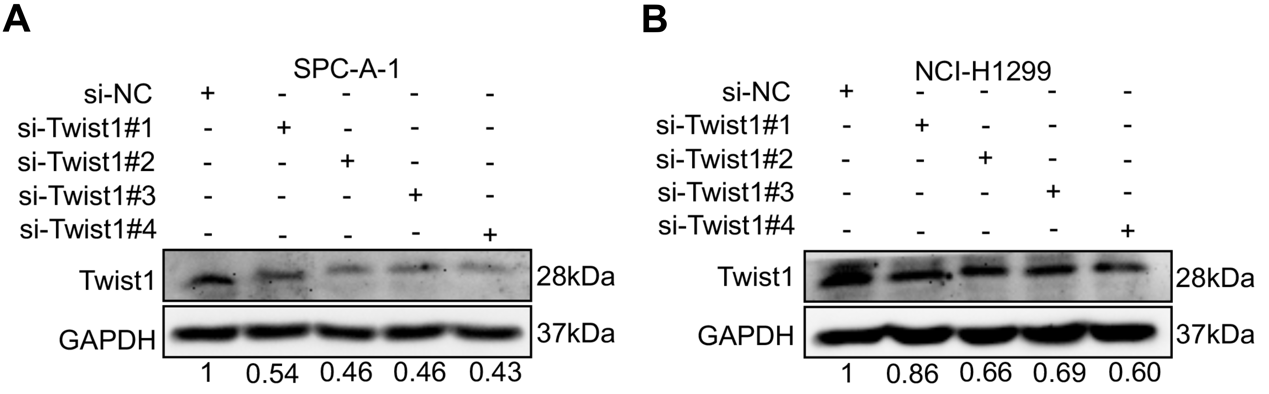


**Figure S3 Knockdown of Twist1 in lung cancer cells.** Western blot analysis of Twist1 protein in SPC-A1 (**A**) and NCI-H1299 (**B**) cells transfected with control siRNA (si-NC) and siRNA to Twist1 (si-Twist1#1, si-Twist1#2, si-Twist1#3, si-Twist1#4).
